# Is it possible to predict the severity of acute appendicitis? Reliability of predictive models based on easily available blood variables

**DOI:** 10.1186/s13017-023-00478-8

**Published:** 2023-01-27

**Authors:** Barza Afzal, Roberto Cirocchi, Aruna Dawani, Jacopo Desiderio, Antonio Di Cintio, Domenico Di Nardo, Federico Farinacci, James Fung, Alessandro Gemini, Lorenzo Guerci, Sen Yin Melina Kam, Svetlana Lakunina, Lee Madi, Stefano Mazzetti, Bakhtiar Nadyrshine, Ola Shams, Maria Chiara Ranucci, Francesco Ricci, Afroza Sharmin, Stefano Trastulli, Tanzela Yasin, Giles Bond-Smith, Giovanni D. Tebala

**Affiliations:** 1 Surgical Emergency Unit, Oxford University Hospital NHS Foundation Trust, Oxford, UK; 2 Digestive and Emergency Surgery Unit, S.Maria Hospital Trust, Terni, Italy

**Keywords:** Acute appendicitis, Appendicectomy, Predictive models, Lymphocyte count, c-reactive protein, Albumin

## Abstract

**Introduction:**

Recent evidence confirms that the treatment of acute appendicitis is not necessarily surgical, and selected patients with uncomplicated appendicitis can benefit from a non-operative management. Unfortunately, no cost-effective test has been proven to be able to effectively predict the degree of appendicular inflammation as yet, therefore, patient selection is too often left to the personal choice of the emergency surgeon. Our paper aims to clarify if basic and readily available blood tests can give reliable prognostic information to build up predictive models to help the decision-making process.

**Methods:**

Clinical notes of 2275 patients who underwent an appendicectomy with a presumptive diagnosis of acute appendicitis were reviewed, taking into consideration basic preoperative blood tests and histology reports on the surgical specimens. Variables were compared with univariate and multivariate analysis, and predictive models were created.

**Results:**

18.2% of patients had a negative appendicectomy, 9.6% had mucosal only inflammation, 53% had transmural inflammation and 19.2% had gangrenous appendicitis. A strong correlation was found between degree of inflammation and lymphocytes count and CRP/Albumin ratio, both at univariate and multivariate analysis. A predictive model to identify cases of gangrenous appendicitis was developed.

**Conclusion:**

Low lymphocyte count and high CRP/Albumin ratio combined into a predictive model may have a role in the selection of patients who deserve appendicectomy instead of non-operative management of acute appendicitis.

## Introduction

Acute appendicitis is one of the most frequent indications for emergency surgical admission [1]. Traditionally, the treatment of acute appendicitis has always been surgical appendicectomy, even if there is good evidence that at least some cases of appendicitis can be treated conservatively. The recent Covid-19 pandemic and the subsequent guidelines to avoid potentially unnecessary surgery during the peak of the pandemic to reduce the workload of our already strained health systems have taught us that a significant percentage of patients with acute appendicitis can be treated conservatively, thus reducing the risks of a surgical operation [2]. Identifying those patients is not as straightforward as it could seem, and failure of conservative management is still quite high [3]. Generally speaking, the efficacy of conservative management depends on the histologic changes of the appendix, being more likely in the early stages of acute appendicitis and in the absence of a faecalith obstructing the lumen of the appendix [4]. However, it is quite difficult to predict the degree of inflammation of the appendix preoperatively, and CT scan has gained popularity as a diagnostic and prognostic tool for acute appendicitis [5]. Unfortunately, CT is not the ideal screening test, as it is expensive and carries a low but discrete risk of radiation-related morbidity. Furthermore, any diagnosis based on single laboratory tests is not as specific as we would need to differentiate complicated vs non-complicated appendicitis and is associated with a non-insignificant risk of false positive. With this study, we tried to build up a predictive model to stratify preoperatively patients with a diagnosis of acute appendicitis to identify those with advanced (or complicated) disease based on easily available laboratory variables.

## Materials and methods

Electronic notes of patients operated of appendicectomy from 1 September 2016 to 31 August 2021 were retrospectively retrieved and analysed as part of an audit of the Surgical Emergency Unit of the Oxford University Hospitals NHS Foundation Trust. Further analysis and discussion have been conducted in collaboration with the Digestive and Emergency Surgery Unit of the Hospital of Terni (Italy).

This audit was approved by the Audit Committee of the Oxford University Hospitals NHS Foundation Trust. Ethical committee approval was not deemed to be necessary as data were collected retrospectively and were all anonymised.

Inclusion criteria were given as follows: age ≥ 16yo, preoperative diagnosis of appendicitis, laparoscopic or open operation. Exclusion criteria were given as follows: age < 16yo, BMI > 35, significant comorbidity (cardiac, liver or renal disease, cancer diagnosis, peripheral vascular disease, immunodeficiency, diabetes, coagulation disorder), pregnancy and appendicectomy as part of another operation. Preoperative clinical and laboratory data were retrieved and recorded into an electronic database (Microsoft Excel for Mac v.16.66.1) along with the histology findings on the operative specimen. Cases with > 20% of incomplete data were excluded. Missing data were excluded listwise.

Patients were divided into four categories according to the histologic findings: 1—no appendicitis, 2—mucosal appendicitis, 3—transmural appendicitis and 4—gangrenous appendicitis (including perforated appendixes).

Continuous variables were first analysed for skewness (− 0.5 to 0.5 is normal distribution) and then compared with the ANalysis Of VAriance test (ANOVA). Subsequently, the variables that gained statistical significance at univariate analysis were introduced into a multinomial (ordinal) logistic regression analysis to identify the independent prognostic factors for the histopathology findings (1, 2, 3 or 4 as above). Subsequently, the same factors were entered into a binomial logistic regression analysis to identify the prognostic factors for “gangrenous appendicitis” vs “non-gangrenous appendicitis” or “non-inflamed appendix” (dependent variable) and to create a predictive model, whose model fit measures and ROC curve were calculated. Sensitivity and specificity of the models were calculated. Starting from the last predictive model (“gangrenous vs non-gangrenous appendicitis”), variables were progressively removed until the maximum specificity of the model was obtained. This allowed the identification of a simplified predictive model for “gangrenous appendicitis”.

Statistical analyses were performed with the applications StatPlus for Mac v.8.0.1.0 and Jamovi v.1.2.9.0. Continuous variables are approximated to the thousandths. *P* values are approximated to the thousandths. *P* values < 0.05 are considered to be significant.

## Results

We retrieved clinical and laboratory data of 2275 patients who had appendicectomy with a preoperative clinical diagnosis of acute appendicitis and fulfilled the inclusion criteria. At histology, 414 (18.2%) did not have any appendicular inflammation, 219 (9.6%) had a mucosal only appendicitis, 1205 (53.0%) had transmural inflammation and 437 (19.2%) had gangrenous appendicitis.

Results of univariate analysis are reported in Table 1. Albumin, CRP, lymphocytes count, neutrophils count, WBC count, CRP/Albumin ratio, CRP/MPV ratio, albumin/MPV ratio and neutrophils/lymphocytes ratio were found to be directly or inversely associated with severity of appendicitis. In particular, a strong correlation was evident between lymphocytes count and degree of inflammation and CRP/Albumin ratio and degree of inflammation.

**Table 1:** Univariate comparison of laboratory variables

Variable	Skewness	No appendicitis	Mucosal appendicitis	Transmural appendicitis	Gangrenous appendicitis	*p*
Albumin (g/dL)	− 0.5	38.732 ± 4.203 38.326 − 39.138	39.516 ± 4.577 38.906 − 40.125	39.665 ± 4.295 39.422 − 39.907	37.460 ± 4.788 37.010 − 37.910	**< 0.001**
ALP (U/L)	3.6	75.886 ± 22.587 73.701 − 78.071	74.849 ± 25.195 71.494 − 78.205	75.157 ± 25.049 73.740 − 76.574	77.039 ± 32.724 73.962 − 80.115	0.599
ALT (U/L)	5.8	21.583 ± 13.480 20.277 − 22.888	25.251 ± 25.896 21.802 − 28.670	23.704 ± 21.113 22.504 − 24.904	22.610 ± 21.527 20.576 − 24.643	0.123
Tot bilirubin (umol/L)	2.8	15.709 ± 10.665 14.679 − 16.741	14.936 ± 9.212 13.709 − 16.163	16.094 ± 11.438 15.447 − 16.741	16.204 ± 10.761 15.192 − 17.215	0.475
Creatinine (umol/L)	6.3	68.656 ± 16.738 67.037 − 70.275	70.562 ± 19.517 67.962 − 73.161	70.115 ± 17.150 69.144 − 71.085	71.420 ± 27.998 68.784 − 74.055	0.239
CRP (mg/dL)	1.9	42.419 ± 60.874 36.538 − 48.300	56.939 ± 73.333 47.172 − 66.705	58.777 ± 70.485 54.793 − 62.761	119.710 ± 104.941 109.843 − 129.576	**< 0.001**
Hb (g/L)	− 0.3	140.562 ± 16.017 139.012 − 142.111	142.205 ± 13.626 140.391 − 144.020	140.726 ± 15.113 139.870 − 141.582	141.165 ± 15.113 139.751 − 142.579	0.547
Lymphocytes (x10^9^L)	1.1	1.685 ± 0.896 1.599 − 1.772	1.705 ± 0.815 1.596 − 1.813	1.603 ± 0.743 1.561 − 1.645	1.478 ± 0.778 1.405 − 1.551	**< 0.001**
MCV (fL)	− 0.6	87.058 ± 4.373 86.635 − 87.481	87.617 ± 5.093 86.939 − 88.296	87.444 ± 4.565 87.185 − 87.702	87.613 ± 4.884 87.154 − 88.073	0.298
MPV (fL)	0.5	10.218 ± 0.840 10.136 − 10.299	10.267 ± 0.921 10.144 − 10.390	10.248 ± 0.892 10.197 − 10.299	10.264 ± 0.897 10.179 − 10.348	0.872
Neutrophils (x10^9^L)	0.3	8.780 ± 4.486 8.346 − 9.213	10.133 ± 4.554 9.527 − 10.740	10.179 ± 4.320 9.934 − 10.423	10.709 ± 4.540 10.281 − 11.136	**< 0.001**
Platelets (x10^9^L)	1.0	253.731 ± 59.12 248.397 − 259.468	258.068 ± 72.617 248.397 − 267.740	253.697 ± 64.262 250.057 − 257.336	252.172 ± 64.281 246.115 − 258.230	0.741
Sodium (mmol/L)	− 1.0	138.322 ± 2.209 138.108 − 138.535	138.434 ± 2.254 138.134 − 138.734	138.179 ± 2.460 138.040 − 138.318	137.980 ± 2.600 137.735 − 138.223	0.080
WBC (x10^9^L)	0.3	12.310 ± 4.667 11.859 − 12.761	13.101 ± 4.543 12.496 − 13.707	12.540 ± 4.392 12.291 − 12.788	13.081 ± 4.750 12.634 − 13.528	**0.027**
CRP/Albumin	2.7	1.208 ± 1.882 1.027 − 1.390	1.569 ± 2.183 1.279 − 1.860	1.609 ± 2.202 1.484 − 1.733	3.476 ± 3.394 3.157 − 3.796	**< 0.001**
CRP/MPV	2.0	5.406 ± 6.639 4.764 − 6.048	6.201 ± 8.110 5.120 − 7.281	6.213 ± 7.821 5.770 − 6.656	9.028 ± 9.060 8.175 − 9.881	**< 0.001**
Albumin/MPV	− 0.1	3.851 ± 0.521 3.801 − 3.902	3.894 ± 0.514 3.826 − 3.963	3.847 ± 0.550 3.816 − 3.878	3.774 ± 0.571 3.720 − 3.828	**0.031**
Neutr/Lymph	2.3	7.238 ± 6.538 6.605 − 7.870	8.122 ± 6.802 7.216 − 9.028	8.726 ± 7.338 8.310 − 9.142	10.191 ± 8.383 9.403 − 10.981	**< 0.001**

Values are reported as mean ± standard deviation and 95% confidence interval. ALP: alkaline phosphatase, ALT: alanine aminotransferase, CRP: C-reactive protein, Hb: haemoglobin, MCV: mean cell volume, MPV: mean platelet volume and WBC: white blood cells. *p* < 0.05 is statistically significant (bold)

**Fig. 1:**
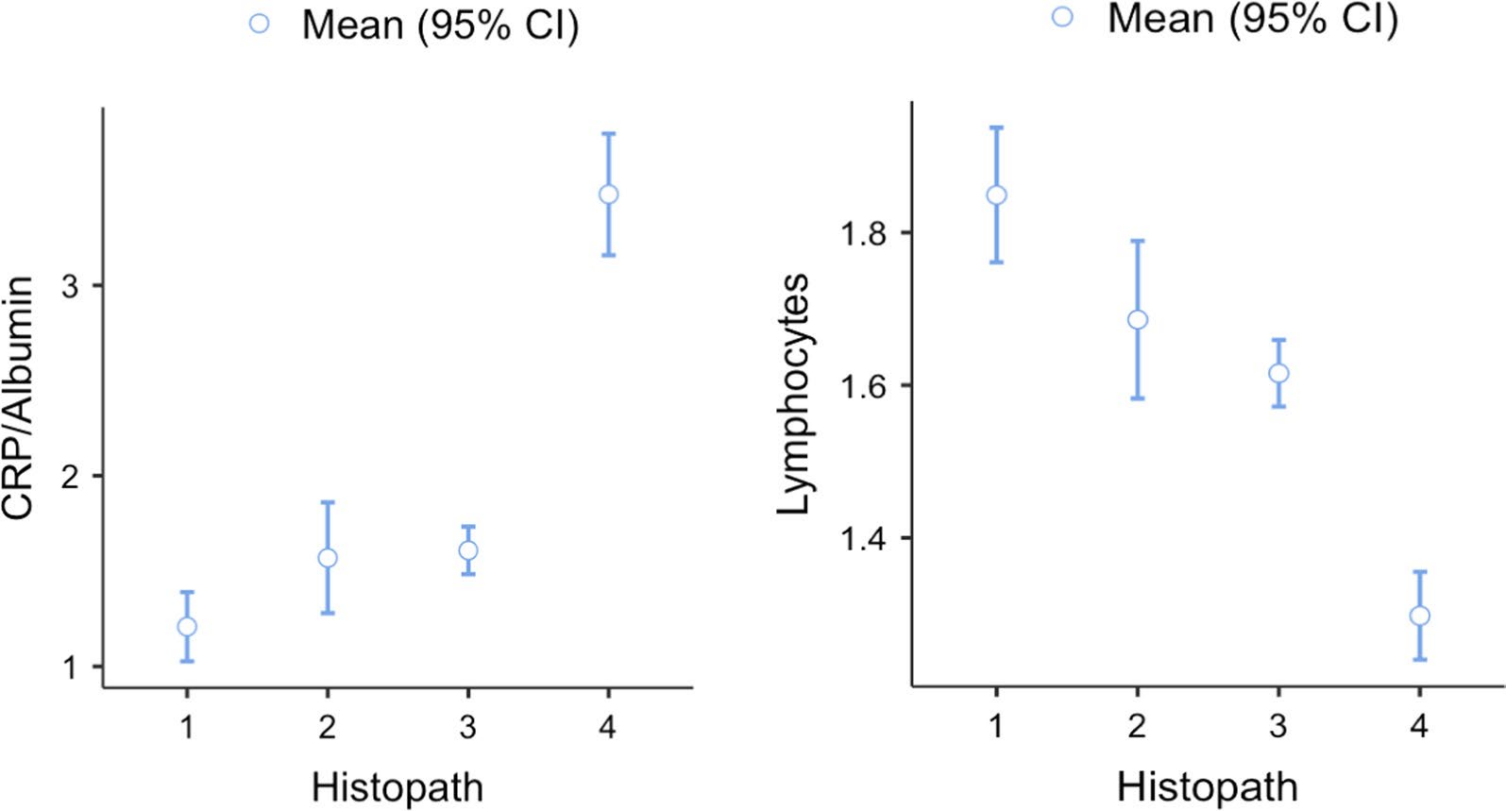
Descriptive plot of lymphocyte count and CRP/Albumin ratio by histopathology group. Histopathology: 1—no inflammation, 2—mucosal inflammation, 3—transmural inflammation and 4—gangrenous appendicitis

These two variables—lymphocyte count and CRP/albumin ratio—resulted particularly abnormal in patients with gangrenous appendicitis (Fig. 1).

**Fig. 2:**
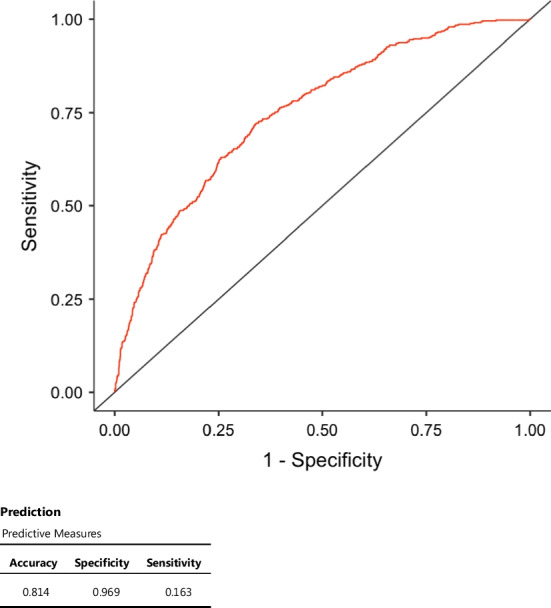
Receiver operating characteristic (ROC) curve of the predictive model for gangrenous appendicitis (see Table 3)

**Fig. 3:**
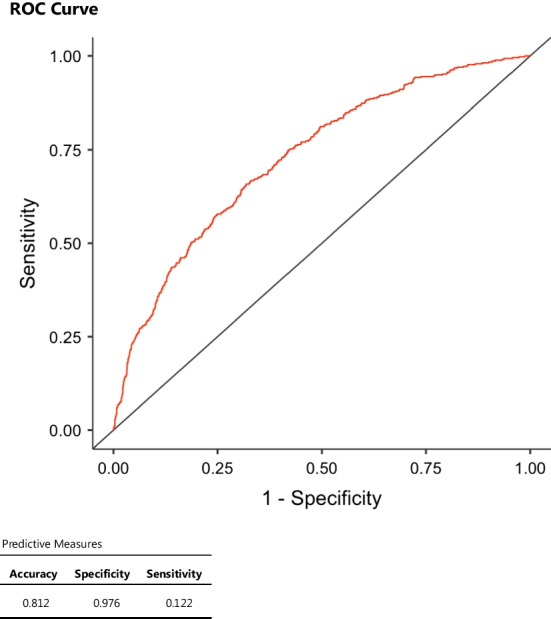
Receiver operating characteristic (ROC) curve of the simplified predictive model for gangrenous appendicitis (see Table 4)

Tables 2, 3 and 4 report the results of multivariate analysis. Figures 2 and 3 show the ROC curve and predictive measures of the predictive models. Of the factors entered into the multivariate analysis, only lymphocyte count and CRP/Albumin ratio resulted to be significantly independent prognostic factors for the severity of inflammation (Table 2). When the dependent variable was “gangrenous appendicitis”, albumin, lymphocyte count, neutrophil count, WBC count and CRP/Albumin ratio were independent prognostic variables (Table 3). The predictive model for “gangrenous appendicitis” has low sensitivity but high specificity (Fig. 2). The simplified model obtained by consecutive regression analyses with progressive elimination of variables (backward stepwise regression) to identify the model with the highest specificity is shown in Table 4 and Fig. 3. However, it must be highlighted that R^2^ for all those models is quite low, from 0.082 to 0.132, which means that only 8–13% of the variability can be explained by the models.

**Table 2:** Multivariate analysis by ordinal logistic regression and model fit measures

Model fit measures						
				Overall Model Test		
Model	Deviance	AIC	*R*^2^_McF_	*χ*^2^	df	*p*
1	4927	4951	0.0821	441	9	< .001

Model coefficients—histopathology					
Predictor	Estimate	SE	*Z*	*p*	Odds ratio
Albumin	− 0.00402	0.01923	− 0.2093	0.834	0.996
CRP	0.00806	0.00665	1.2126	0.225	1.008
Lymphocytes	− 0.40651	0.15749	− 2.5811	0.010	0.666
Neutrophils	0.00697	0.12948	0.0538	0.957	1.007
WBC	0.12731	0.12169	1.0462	0.295	1.136
CRP/Albumin	− 0.20773	0.08694	− 2.3895	0.017	0.812
CRP/MPV	0.04142	0.06285	0.6590	0.510	1.042
Albumin/MPV	− 0.08187	0.15347	− 0.5335	0.594	0.921
Neutr/Lymph	− 0.00689	0.01012	− 0.6809	0.496	0.993

The dependent variable 'Histopathology' has the following order: 1 | 2 | 3 | 4

Dependent variable: histopathology findings (1: no inflammation, 2: mucosal appendicitis, 3: transmural appendicitis and 4: gangrenous appendicitis). Only variables that were significant at univariate analysis were introduced into the multivariate analysis. CRP: C-reactive protein, MPV: mean platelet volume and WBC: white blood cells

**Table 3:** Multivariate analysis by binomial logistic regression and model fit measures

Model fit measures						
				Overall model test		
Model	Deviance	AIC	R^2^_McF_	χ^2^	df	*p*
1	1923	1943	0.132	291	9	< .001

model coefficients—gangrenous appendicitis					
Predictor	Estimate	SE	Z	*p*	Odds ratio
Intercept	0.72593	0.73657	0.9856	0.324	2.067
Albumin	− 0.07223	0.02966	− 2.4357	0.015	0.930
CRP	0.00994	0.00756	1.3148	0.189	1.010
Lymphocytes	− 1.09416	0.22605	− 4.8403	< .001	0.335
Neutrophils	− 0.41807	0.16457	− 2.5405	0.011	0.658
WBC	0.47877	0.15503	3.0882	0.002	1.614
CRP/Albumin	− 0.32336	0.11364	− 2.8455	0.004	0.724
CRP/MPV	0.05080	0.07029	0.7227	0.470	1.052
Albumin/MPV	− 0.01318	0.24328	− 0.0542	0.957	0.987
Neutr/Lymph	− 0.00422	0.01296	− 0.3256	0.745	0.996

Estimates represent the log odds of "Gangrenous Appendicitis = 1" versus "Gangrenous Appendicitis = 0"

Dependent variable: gangrenous appendicitis. Only variables that were significant at univariate analysis were introduced into the multivariate analysis. CRP: C-reactive protein, MPV: mean platelet volume and WBC: white blood cells

**Table 4:** Simplified predictive model for gangrenous appendicitis

Model fit measures			
Model	Deviance	AIC	*R*^2^_McF_
1	1996	2002	0.101

Model coefficients—gangrenous appendicitis					
Predictor	Estimate	SE	*Z*	*p*	Odds ratio
Intercept	− 1.130	0.1504	− 7.51	< .001	0.323
Lymphocytes	− 0.557	0.0870	− 6.40	< .001	0.573
CRP/Albumin	0.227	0.0208	10.90	< .001	1.255

Prediction		
Predictive measures		
Accuracy	Specificity	Sensitivity
0.812	0.976	0.122

Estimates represent the log odds of "Gangrenous Appendicitis = 1" versus "Gangrenous Appendicitis = 0"

The cut-off value is set to 0.5

CRP: C-reactive protein

## Discussion

Acute appendicitis is one of the most common reasons for emergency surgical admission [1]. In the past, almost invariably, a clinical diagnosis of acute appendicitis, often but not always associated with laboratory confirmation (leucocytosis), led to a surgical operation of appendicectomy. As a consequence, the rate of negative appendicectomies (i.e. whose macroscopic or pathologic examination did not find any sign of inflammation) was quite high. The advent of laparoscopy, with its diagnostic capabilities, should have reduced the rate of negative appendicectomies [6, 7] but this has never been definitely proven [8]. In actual facts, a certain number of cases where symptoms would suggest acute appendicitis were due to other diseases such as tubo-ovarian inflammation, ectopic pregnancy, active ileitis or colitis or simply to mesenteric adenopathy, among the others. In some cases, even laparoscopic exploration is not able to find the cause of right iliac fossa pain. Guidelines on the diagnosis and treatment of acute pain in the right iliac fossa are not always consistent and there are still areas for discussion. Laparoscopic exploration is nowadays considered the gold standard for diagnostic and therapeutic purposes [6, 7]. While the indication for appendicectomy is straightforward in case of macroscopically confirmed acute appendicitis, it has been suggested not to remove the appendix if another possible cause for pain has been detected and possibly treated [9]. The issue remains in those cases where abdominal exploration does not reveal any abnormality in a patient with clear symptoms of appendicitis. The European Association of Endoscopic Surgery Guidelines and the World Society of Emergency Surgery Guidelines suggest removing the appendix anyway, considering the possibility of a mucosal only appendicitis which is not visible from the serosal surface [7, 10], but this approach may be considered an overtreatment and expose to unnecessary, albeit low, risk of complications in cases of innocent appendixes [11]. Ultrasound scan is rarely diagnostic of acute appendicitis, and the rate of false negatives is quite high [10]. Nonetheless, most surgeons still consider blood tests and abdominal USS the basis of the diagnosis of acute appendicitis. To reduce the risk of unnecessary appendicectomies, some Authors and guidelines suggested a liberal use of preoperative CT scan [7]. However, it is well known that also CT scan can be associated with false negative results and it is not immune from radiation-associated risks, in particular in young people [12]. Magnetic resonance imaging (MRI) is expensive and time-consuming, and its real indications in emergency are usually limited to pregnant patients and in case of suspected inflammatory bowel, unless new MRI protocols are developed and implemented specifically for the emergency evaluation of right iliac fossa pain [13]. Up to date, no reliable test, either imaging or laboratory based, has been found to be able to accurately predict the presence of acute appendicitis.

On the other side of the coin, the recent Covid-19 pandemic has completely changed our attitude towards some urgent conditions, teaching us that in some cases refraining from surgery can be the safer option [14]. This has been the case with acute right iliac fossa pain [15]. More recent evidence suggest that most cases of suspected acute appendicitis can have a non-operative management [7], because they can be due either to mild appendicitis that resolves spontaneously or to some other ailment not deserving a surgical operation, such as pelvic inflammatory disease [2]. Some evidence seems to suggest that any non-complicated acute appendicitis can be treated non-operatively unless the presence of a faecalith in the lumen of the appendix prevents its drainage and may increase the risk of mucocele or perforation [4]. Advanced imaging—either CT or MRI—can rule out or confirm the presence of an obstructed appendix, but, as already mentioned, those investigations are not without risks or downsides. Some other evidence confirms that an appendicectomy is indicated only in gangrenous or perforated appendicitis, while non-complicated appendicitis can be treated conservatively, even if one fifth of patients treated non-operatively require an appendicectomy within 30 days from the first diagnosis [16]. Unfortunately, at the moment there is no test that has a clinically significant predictive value for gangrenous appendicitis or that can help us foresee the extent of appendicular inflammation, if any.

Thus, the need to develop a reliable, quick, safe and easily available test to select the patients with acute right iliac fossa pain who may benefit of a surgical operation arises. Any patient seen in the Emergency Department with acute abdominal pain gets at least a blood sample for basic analysis. If correctly interpreted possibly in an aggregated fashion, those values can be highly informative and may help predict the degree of inflammation. Traditionally, white blood cells (WBC) count has been considered a marker of inflammation, along with C-reactive protein (CRP) [7], but WBC and CRP are not highly specific of the degree of inflammation and cannot be used for precise patient selection [17]. Other possible markers of inflammation, such as procalcitonin and interleukin, are not routinely checked in emergency [18]. The Alvarado score [19] and the Appendicitis Inflammatory Response (AIR) Score [20] were laudable attempts at enhancing the diagnostic efficacy but they are not completely reliable and not widely used [7, 21]. The Alvarado score has high sensitivity (99%) to rule out appendicitis when its score is < 5, but its overall sensitivity is between 57 and 93%, with specificity ranging from 81 to 100% [22]. An AIR score ≥ 5 is highly sensitive for appendicitis (90%), but overall sensitivity ranges from 78 to 92% and specificity from 63 to 97% [22]. To overcome the uncertainties associated with these scoring systems, various combinations of imaging and laboratory tests have been proposed, but diagnostic laparoscopy without imaging is still considered a viable option in particular in patients who scored high at Alvarado or AIR [22]. Augustin et al. [23] proposed the Appendicitis Tri-Modal Prediction Score (ATMOS) for the differential diagnosis of right iliac fossa pain in pregnant women, but it has not been externally validated yet.

Our study on a relevant number of cases was aimed at identifying any prognostic factor with significant predictive value among the most common variables easily available from any laboratory at the admission of the patient. Although several variables were associated with histopathology findings at univariate analysis, multivariate analysis confirmed that only lymphocyte count and CRP/Albumin ratio were independently associated with the degree of inflammation. Both variables’ odds ratios are below 1, which means they both have an inverse correlation. In other words, it looks like low lymphocyte count and low CRP/Albumin ratio can be predictive of the severity of inflammation. However, only 8% of the variability of severity of inflammation can be predicted by this model.

Low lymphocyte count is often associated with high neutrophil count; therefore it would seem logical that in acute appendicitis, neutrophils to lymphocyte ratio should be increased. In actual facts, neutrophils/lymphocytes ratio showed a positive correlation with the degree of inflammation at univariate analysis, but this was not confirmed at regression analysis. In other terms, our study showed that neutrophils/lymphocytes ratio may not be reliable to select patients with gangrenous appendicitis who may need an emergency surgical operation.

On the contrary, low lymphocyte count has been identified as an independent prognostic variable who significantly correlates to the degree of inflammation.

Similarly, while CRP per se did not qualify as a diagnostic tool in our study, the CRP to Albumin ratio is significantly and independently associated with severity of appendicitis. CRP is a well-known marker of inflammation, but it is not specific. Albumin is often erroneously considered a nutritional marker but, on the contrary, it has been demonstrated to be much more reliable as an inverse inflammatory marker [24]. Therefore, CRP/Albumin should be directly correlated with the degree of inflammation, as high CRP and low Albumin are both linked to inflammation. In fact, our simplified predictive model considers only low lymphocyte count and high CRP to albumin ratio to significantly correlate with the presence of gangrenous appendicitis. This model has high accuracy (81%) and high specificity (98%).

In other words, low lymphocyte count and CRP/Albumin can be used as markers to select those patients with acute right iliac fossa pain who would benefit from an operation of appendicectomy, i.e. those with gangrenous appendicitis, among those who have a clinical diagnosis of acute appendicitis. Patients with a different laboratory pattern may be considered for non-operative management.

Strengths of this paper are the conspicuous sample size and the fact that an easy predictive model was built up from easily available blood variables.

However, there is also a significant limitation, as the models have low *R*^2^, making us infer that basic blood tests may not be specific enough to have a clinically significant prognostic power. This consideration may prompt someone to propose the use of much more expensive and not readily available tests, such as IL-2, to allow an accurate selection of patients, but acute appendicitis is a common presumptive diagnosis in emergency surgery and a condition with low social impact; for this reason, the expenses associated with high-priced tests may not be justified and sustainable. More research may be needed to see if the “clinical acumen” of experienced surgeons may be more reliable than any laboratory or imaging test in the selection of patients with acute right iliac fossa pain.

However, the reliability of the model must be externally validated on large series.

In conclusion, low lymphocyte count and high CRP to albumin ratio can have a role in the selection of patients with suspected acute appendicitis who may deserve a surgical operation, but other predictive models, possibly incorporating clinical examination, imaging and blood tests, should be considered to reach clinical relevance.

## Data Availability

The dataset generated and analysed during the current study is available from the corresponding author upon reasonable request.

## Abbreviations

CRP: C-reactive protein
Hb: Haemoglobin
MCV: Mean cell volume
MPV: Mean platelet volume
WBC: White blood cells
